# Antioxidant premedication reduces mitochondrial and nuclear DNA damage repair in interventional physicians performing X-ray-guided angiography procedures: a pilot clinical intervention study

**DOI:** 10.1186/s42155-026-00777-6

**Published:** 2026-09-22

**Authors:** Kieran Murphy, Thomas Lindsay, Kongteng Tan, Sean Lau, Gavin Elias, Kieran Luke, Josh Nederveen, Donald Xhuti, Mahek Minhas, Benjamin Lok, Shusen Zhu, Gaylene Pron, Mark Tarnopolsky

**Affiliations:** 1 Department of Medical Imaging, Toronto Western Hospital, University Health Network, 399 Bathurst St., 3rd Fl., Toronto, ON M5T2S8 Canada; 2 Division Vascular Surgery, Toronto General Hospital, University Health Network, 200 Elizabeth St., 6EN-228, Toronto, ON M5G2C4 Canada; 3 Joint Department of Medical Imaging, University Health Network, 700 University Avenue, 2nd Fl., Toronto, ON M5G1X6 Canada; 4 Joint Department of Medical Imaging, University of Toronto, 263 McCaul Street, 4th Fl., Toronto, ON M5T1W7 Canada; 5 Department of Kinesiology, Faculty of Science, McMaster University, 1280 Main St. W., Hamilton, ON L8S4K1 Canada; 6 Faculty Health Sciences, Medical Sciences Graduate Program, McMaster University, 1200 Main St. W., Hamilton, ON L8N3Z5 Canada; 7 Radiation Medicine Program, Princess Margaret Cancer Center, 610 University Avenue, Toronto, ON M5G2M9 Canada; 8 Institute Health Policy Management and Evaluation, University of Toronto, Health Sciences Bldg., 155 College St., Toronto, ON M5T3M7 Canada; 9 Department of Pediatrics, McMaster Children’s Hospital, 1200 Main St. W., Hamilton, ON L8N3Z5 Canada

**Keywords:** Antioxidants, DNA damage response, Ionizing radiation, Mitochondrial DNA, Oxidative stress, Nuclear DNA

## Abstract

**Background:**

To examine effects of naturally occurring antioxidants on DNA damage repair acute responses of ionizing radiation sustained by interventional physicians during angiography.

**Methods:**

The study cohort included 20 interventional physicians (19 males, 1 female; mean age: 45.4 ± 13.2 years) performing angiography fluoroscopically guided procedures. Participants were allocated to a treatment group receiving oral antioxidants (*N* = 10/group) before starting day cases or to an untreated control group performing procedures without antioxidants (*N* = 10/group). mRNA biomarkers of nuclear and mitochondrial DNA injury repair response in peripheral blood lymphocytes were assessed before and after workday.

**Results:**

Baseline DNA repair biomarkers and procedural fluoroscopic exposures were similar between the study groups. Individual pre-post changes in MRNA levels were not significant for any biomarkers in the antioxidant-treated group but were significantly increased in the untreated control group for *P53* (*P* = 0.006), *NF-κB* (*P* = 0.002), and *COX4i1* (*P* = 0.004). Between-group differences in post-exposure gene expression were significantly (*P* = 0.024) higher with *P53* being 78% higher in the untreated versus the treated group and *COX4i1* levels also significantly (*P* = 0.028) higher by 46% in the untreated group. Absolute change in the *STAT3* mRNA level was higher by 54% in the untreated group but between-group difference was not significant (*P* = 0.066). There was a lower burden of *NF-κB* mRNA levels of inflammation in the treatment than the untreated group (18 ± 8% versus 60 ± 15%) with significant between-group difference (*P* = 0.020).

**Conclusions:**

Significant elevations in peripheral blood lymphocytes of mRNA biomarkers of nuclear and mitochondrial DNA injury repair response occurred in untreated interventional physicians exposed to ionizing radiation during fluoroscopically guided interventions. In physicians pretreated with a novel antioxidant formulation, biomarkers of DNA repair responses were significantly attenuated suggesting a decreased acute IR-induced oxidative stress-mediated DNA damage response.

**Supplementary Information:**

The online version contains supplementary material available at https://doi.org/10.1186/s42155-026-00777-6.

## Introduction

Ionizing radiation (IR) adversely impacts human health and has implications for diverse occupational groups [1]. Hospital medical staff, however, represent the largest occupationally chronically exposed workers to low-dose IR [2]. Therapeutic procedures involving X-ray guidance, because of longer exposure durations, can result in even higher radiation exposures for operators performing the studies as well as for patients [3]. Although the per-case exposures may be low and despite risk reduction measures like lead aprons and thyroid collars, interventional physicians can accumulate lifetime radiation exposure in the range of 50 to 200 mSv depending on their practice [4, 5]. These levels could be beyond the International Commission on Radiation Protections (ICRP) regulatory maximum exposure levels of 100 mSv in a 5-year consecutive interval [6].

Ionizing radiation can cause stochastic effects involving DNA damage and genetic alterations increasing cancer risk and is designated a known human carcinogen by the International Agency for Research on Cancer. A recent systematic review [7] on radiation-induced DNA damage and cancer risk for endovascular operators reported an increased risk of cancer particularly for radiation-sensitive tumors such as leukemia and thyroid cancers and in unprotected areas such as the skin and brain. A study investigating brain cancer in interventional physicians found that 85% of identified tumors were left-sided likely attributable to physician positioning relative to the X-ray tube exposing the left side of the head to more IR [8]. Additionally, deterministic effects involving direct tissue damage such as a significantly increased risk of cataracts, especially for interventional cardiologists exposed to IR, have been reported in a systematic review of 21 studies [9]. Although studies evaluating risks of cardiovascular diseases with exposure to low-dose IR have been inconsistent, the ICRP in 2011 listed diseases of the circulatory system to be a radiation health hazard and recommended dose thresholds [10].

Ionizing radiation causes the majority of DNA damage by cellular oxidative stress resulting from unmanaged levels of reactive oxygen (ROS) and nitrogen (RNS) species. The more that DNA damage is repaired or removed quickly, the less likely it is to become a genetic mutation, a key event in the initiation and development of adverse health consequences including cancer [11]. Genomic stability therefore depends on cellular protective repair responses to DNA damaging exposures such as IR [12].

Antioxidants are known to affect biological systems by reducing DNA damage from radiation and may therefore have radioprotective benefits to interventional physicians during procedures [13]. To date, no studies have evaluated the radioprotective effects of naturally occurring antioxidants administered to interventional physicians prior to procedures. The present pilot clinical study therefore sought to determine whether a multi-ingredient antioxidant supplement would be radioprotective to interventional physicians performing fluoroscopically guided procedures in the angiography suite.

## Materials and methods

### Study design and participants

This pilot clinical study received institutional research ethics board approval from a university-affiliated teaching hospital. Approval was obtained prior to study initiation and all participants gave informed consent. A convenience sample was obtained and as this study was a first in this area, there were no priors to base a sample calculation. All eligibility criteria, laboratory protocols, and outcome measures were established before study initiation and participant recruitment. After study commencement, no changes occurred to the study protocol including trial eligibility, intervention, or subsequent biospecimen collection or processing. Analysis of biospecimens were conducted by staff anonymously to the study antioxidant assignment.

We recruited currently practicing physicians specializing in the fields of interventional radiology, interventional neuroradiology, or vascular surgery frequently performing angiography fluoroscopically guided procedures. Inclusion criteria included staff interventionalists working a minimum 3 days a week in hospital interventional suites. Exclusion criteria included those with known hypersensitivity to any ingredients of DNA Halo® (Cora Nutraceuticals Inc., Toronto, Ontario, Canada) the antioxidant formulation administered in the study or those having undergone chemotherapy or radiation therapy within 6 months prior to enrollment. Informed consent was obtained from all participants and were recruited between Oct 1, 2022 and December 31, 2022.

During the study period, all participants continued their typical involvement in angiography fluoroscopically guided procedures and the study involved a day of their involvement in order to capture typical daily levels of radiation exposure for interventional procedures. Participants were sequentially allocated to two equally sized groups (*N* = 10/group). The antioxidant treatment group took two oral antioxidant capsules in the morning 1 h prior to starting their imaging-guided procedures while the untreated control group did not receive any intervention before starting their procedures. All procedures were performed in the angiography suite under fluoroscopic guidance and all operators wore lead aprons and thyroid collars during procedures (Ultraray Medical, Oakville, Ontario, Canada). Real-time radiation dosimeters were not available for operator dose exposures. Fluoroscopic indices of cumulative procedural radiation dose and duration were extracted from the radiation dose summary report generated by the fluoroscopic units for two time periods—the day of the study period and 1 day in the-work week prior to the study. The medicinal ingredients per capsule (DNA Halo®) included vitamin C (100 mg), vitamin E (67 mg), vitamin B12 (25 mcg), folate (200 mcg), selenium (50 mcg), quercetin (100 mg), CoQ10 (67 mg), DL-alpha lipoic acid (67 mg), astaxanthin (2 mg), and zeaxanthin (1.25 mg). Several of the antioxidants, CoQ10, vitamin E, and alpha-lipoic acid were included in the formulation due to their demonstrated effects to improve mitochondrial function in patients with mitochondrial disease [14].

### Sample collection and preparation

Collection and preparation of peripheral blood lymphocytes for the study were as follows. Both study groups had blood drawn in the morning (1 h before starting cases and before receiving antioxidant premedication) and in the evening after completing cases that same day. Further blood draws were not taken. From each participant, one sample of 5–8 ml of whole blood later analyzed in triplicate was drawn peripherally into EDTA-treated tubes and sent on ice to the processing lab. Within 2 h of blood withdrawal, the blood was spun with a Beckman Coulter centrifuge at 3000 × g for 10 min at 4 °C. After gently removing the plasma, 0.3–0.5 ml of the buffy coat was collected to a sterile 1.5-ml Eppendorf tube and stored at − 80 °C. Frozen stored samples underwent further preparation and analysis with laboratory staff blinded to antioxidant assignment. The laboratory protocols for RNA isolation, complementary DNA (cDNA) synthesis, and reverse transcription-polymerase chain reaction analysis (RT-PCR) are documented in Appendix 1.

### mRNA biomarkers and analysis

The selection of biomarkers for the study outcomes was based on their key roles in the IR-induced DNA damage repair response and inflammatory pathways. A primary acute nuclear DNA response outcome measurement for DNA damage repair for the study included messenger RNA (mRNA) levels of *P53* that activates signaling pathways in the critical DNA damage detection and repair response pathways [15]. Other DNA repair biomarkers included mRNA levels of *STAT3* [16] and *COX4iL* [17] as well as *NF-κB* [18], a key marker of the inflammatory process. Primary and secondary mRNA biomarkers along with TaqMan probe IDs (Thermo Fisher Scientific) were quantified in each sample and are detailed in Appendix 2. Specifically, mRNA expression levels were quantified and normalized to the reference gene *B2M* (*beta-2-microglobulin*, Hs00187842_m1) via the 2^−ΔCt^ method. The cycle threshold (Ct) scores of mRNAs of interest were compared with the Ct scores of the reference gene B2M, as previously described [19]. Normalized values were then expressed as fold changes to the PRE time point samples.

Statistical analysis was done using the paired two-tailed *t*-tests comparing mRNA expression levels between PRE and POST time points in a within-subject fashion for the *antioxidant* and untreated *control* groups. Unpaired *t*-tests were used to compare the post-radiation exposure magnitude of PRE-to-POST expression change between the groups. PRISM version 7.0 software was used to enter and analyze real-time PCR data. Descriptive variables (mean and standard error), statistics (*t*-tests), and figure plots were also calculated with PRISM version 7.0 software. Statistical significance was set at *P* = 0.05 or less.

## Results

We conducted a pilot controlled clinical study with 20 interventional physicians (mean age: 45.4 ± 13.2 years; 19 males, 1 female). The mean age of participants in the treated group did not differ from those in the untreated control group, all participants were non-smokers, and the majority were male (Table 1). The study participants all had over 5 years of experience performing angiography image-guided procedures at the same institute and in the same procedure rooms. All participants maintained their original study group allotment and PRE and POST blood samples were collected from all participants. Usable samples were analyzed from ten *control* participants and nine *antioxidant* participants as there was insufficient material for one antioxidant participant.

**Table 1:** Baseline characteristics of antioxidant-treated and untreated study participants

	Antioxidant-treated participants	Untreated participants	*P* value
Age, mean ± SD	50.7 ± 16.34	41.2 ± 8.81	0.128
Smoking habit, no:yes	10:0	10:0	–
mRNA biomarker peripheral lymphocyte levels, mean ± SD			
COX4i1	0.272 ± 0.130	0.191 ± 0.058	0.089
*NF-kB*	0.018 ± 0.006	0.012 ± 0.005	0.032
*P53*	0.035 ± 0.022	0.020 ± 0.010	0.066
*STAT3*	0.013 ± 0.005	0.008 ± 0.005	0.061

*Abbreviations*: *COX4i1* cytochrome c oxidase subunit 4i1, *NF-kB *nuclear factor kappa-light-chain-enhancer of activated B cells, *P53* transformation-related protein 53, *STAT3 *signal transducer and activator of transcription. *P* values based on unpaired *t*-tests

**Fig. 1:**
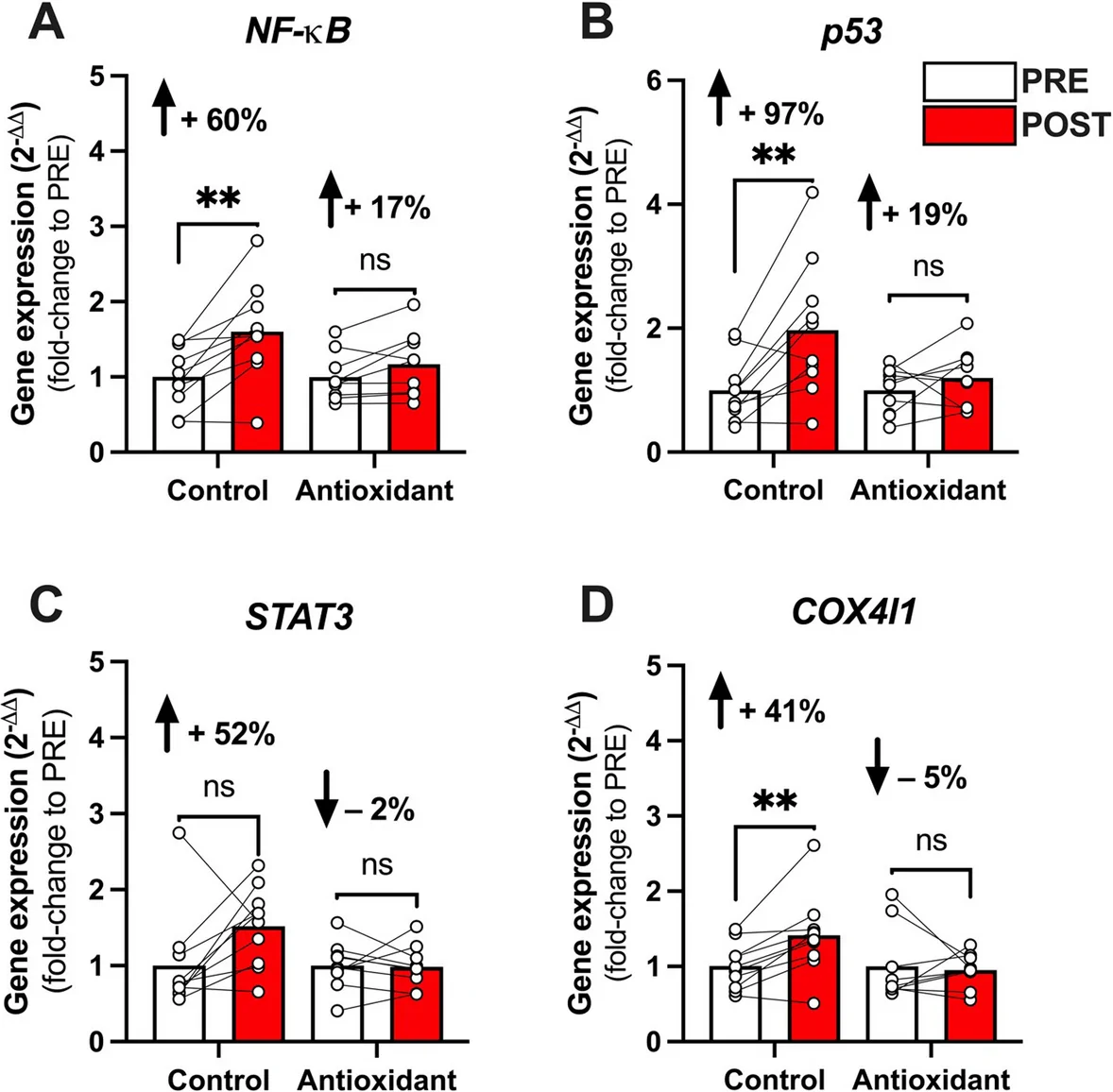
Effects of antioxidant premedication on individual PRE-POST changes in DNA injury repair biomarkers in response to ionizing radiation exposure. Individual gene expression levels of **A** *NF-κB*, **B** *P53*, **C** *STAT3*, and **D** *COX4I1* in peripheral lymphocytes collected from physicians before (PRE) and after (POST) exposure to ionizing radiation while performing fluoroscopically guided procedures on a single day. The antioxidant group received oral antioxidant premedication prior to starting procedures, while the control group did not. ns, not statistically significant (*P* > 0.05); **P < 0.01

Baseline gene expression levels of the target biomarkers in peripheral lymphocytes were not significantly different between the study groups, except for a higher baseline level of NF-*κ*B (*P* = 0.032) in the antioxidant-treated group (Table 1). The fluoroscopy duration and dose during procedures on the test day and on a day in the prior week also did not differ between the study groups (Table 2). Individual PRE-POST changes and significance in gene expression levels for participants in the *control group* and in the *antioxidant group* are detailed in Fig. 1.

**Table 2:** Fluoroscopic exposures during angiographic procedures by antioxidant-treated and untreated participants

	Antioxidant-treated participants	Untreated participants	*P* value
Fluoroscopic exposures one-work week prior to antioxidant trial			
Total fluoroscopic time (minutes, mean ± SD)	71.5 ± 50.468	77.7 ± 47.854	0.786
Total cumulative air kerma (mGy, mean ± SD)	2365.4 ± 2298.286	2135.1 ± 1520.067	0.798
Fluoroscopic exposures during the day of antioxidant trial			
Total fluoroscopic time (minutes, mean ± SD)	51.3 ± 66.132	33.4 ± 30.437	0.476
Total cumulative air kerma (mGy, mean ± SD)	1291.3 ± 1955.393	1283.8 ± 1666.278	0.993

For *NF-κB* mRNA levels, participants in the untreated c*ontrol group* experienced a significant 60 ± 17% (mean ± standard deviation) increase (*P* = 0.002) from the PRE and POST time points, whereas the *antioxidant group* show a lower burden of inflammation with a 17 ± 8% increase (*P* = 0.062) (Fig. 1A). The change in post-radiation exposure inflammatory biomarker was significantly different between groups (*P* = 0.020).

For *P53* expression, the *control group* experienced a significant 97 ± 27% increase in P53 expression between PRE and POST time points (*P* = 0.006), while the *antioxidant group* experienced an attenuated 19 ± 17% increase (*P* = 0.406) (Fig. 1B). The between-group difference in post-exposure *P53* expression changes was significant (*P* = 0.024).

*STAT3* mRNA levels increased by 52 ± 24% in the *control group* (*P* = 0.057) and did not change (− 2 ± 11%) in the *antioxidant group* (*P* = 0.892). The magnitude of change was not significant between study groups (*P* = 0.066) (Fig. 1C).

*COX4i1* levels increased by 41 ± 11% (*P* = 0.004) in the *control group* and did not change (− 5 ± 16%, *P* = 0.760) in the *antioxidant group*. The between-group difference in this post-exposure marker elevation was significant (*P* = 0.028) (Fig. 1D).

## Discussion

The study was a prospective clinical study comparing DNA repair biomarkers in interventional physicians performing fluoroscopically guided interventions pretreated with an antioxidant formulation with those who were untreated. The main findings included significant elevations in DNA repair biomarkers in the untreated but not the antioxidant-treated participants. The study groups were comparable in sex, smoking status, and years of experience working in the same environment. Validated DNA repair biomarkers and up-to-date laboratory techniques were employed to assess cellular DNA damage repair responses to IR. In particular, baseline levels of the key DNA repair biomarkers and procedural fluoroscopic exposures were similar between the study groups.

We conducted this current study as no prior studies have evaluated the radioprotective effects of naturally occurring antioxidants to low-dose IR despite substantial systematic review evidence documenting cellular oxidative stress and DNA damage in interventional physicians [2, 7, 20]. The following discussion involves contextual information regarding protective mechanisms of antioxidants, linkages of oxidative stress and related DNA damage with IR exposures, and rationale for the study antioxidant formulation.

### Ionizing radiation and DNA damage

Most damage from IR occurs because of the radiolysis of water the main component of tissues resulting in the production of unstable ROS and RNS species if unmanaged can cause oxidative damage to proteins, lipids, and DNA [21]. Because cells normally continuously produce ROS, particularly by the mitochondria, the body has evolved a complex system of antioxidant enzymes and non-enzymes to prevent overloading the system referred to as oxidative stress linked to DNA damage and multiple pathological conditions [22].

Occurrences of DNA damage with low-dose IR exposure have been reported for interventional physicians in several cohort studies [23–25]. Increased cellular oxidative stress along with DNA damage has also been reported in interventional physicians exposed to low-dose IR [26]. Patients undergoing CT examinations or angiographic procedures have also had increased occurrences of DNA damage [27, 28] and both DNA damage and oxidative stress have been reported in patients undergoing diagnostic enhanced low-dose CT [29]. Because oxidative stress and DNA damage occurred at IR exposure levels in these studies that were below limits for occupational exposure (20 mSv per year) established by the ICRP [6], antioxidant supplements that might be radioprotective to IR exposure have been recommended [30, 31].

### DNA damage and genetic influence of polymorphisms of DNA damage repair genes

Concerns have been raised as to whether interventional physicians should be genetically screened as some may be at higher risk of radiation-induced cancer [32]*.* Variability in individual subjects’ response to IR noted in our study (Fig. 1A–D) has been reported by others [33, 34]. The DNA damage repair response system has a key role in maintaining genomic integrity and deficiencies in repair functions involving a range of genes and proteins in signaling pathway and functional alterations in DNA repair capacity [35]. Inherited polymorphisms in DNA repair genes have been proposed to affect the DNA damage response to IR [33] and these effects have been reported in interventional cardiologists [36]. The influence of genetics on cancer risk and radiation exposure has been summarized as “cancer occurs at lower doses in high risk individuals than in low risk individuals” [37].

### Evidence for radioprotective properties of antioxidants

Our study employed antioxidants as a possible protective mechanism to IR as these effects at the cellular level have been established in several previous reviews [38, 39]. There is also substantial evidence for a wide array of antioxidant compounds with protective properties to IR [40]. Several in vitro studies [41–43] evaluating these effects have reported reduced DNA damage (double strand breaks (DSB)) in lymphocytes pre-incubated with various combinations of naturally occurring antioxidants prior to irradiation (10 mGy). Levels of radioprotection in the studies depended on the antioxidant employed and reductions in DSBs ranged from 42 to 68% and 65 to 74% [41], 58% [43], and 43%% [44]. The levels of reduced DNA damage for the in vitro studies were similar to the reduced levels of DNA repair biomarkers that occurred in our study with interventional physicians exposed to IR during procedures.

Patients pretreated with antioxidants and undergoing different CT evaluations have also shown significant reductions in DNA damage in lymphocytes compared to untreated patients [45–47]. The Jafarpour et al. study [45] involving a 337 ± 116 mGy radiation dose reported a 22% reduction in DNA damage and the Stehli et al. study (Stehli, 2014 #20) involving a radiation dose of 29 mSv (range 15.5–52.6 mSv) during cardiac CT angiography reported reductions of DNA damage varying from 43 to 87% with different antioxidants. The Velauthapillai et al. study [47] involving antioxidants administered to patients prior to undergoing ^99m^Tc MDP bone scans (798 mBq) for cancer staging reported a 48% reduction in lymphocyte DSBs. The only study [48] investigating radiology workers involved antioxidants administered to X-ray technicians who experienced significantly decreased levels of plasma and erythrocyte oxidative stress (decreased lipid peroxidation) prior to low-dose IR exposure (0.21–0.32 mSv). Although this outcome measure differed from our study, the observation that oxidative stress levels in antioxidant-treated participants remained virtually unchanged paralleled similar results in our study where DNA repair biomarkers were attenuated over baseline in antioxidant-treated participants. Cigarette smoke also significantly increased oxidative stress and was significantly higher in smokers than non-smokers in the untreated X-ray group. Although all the participants in our study were non-smokers, smoking status may be an important factor for other studies.

### Rational for antioxidant formulation in the present study

The antioxidant formulation in our study was employed for several reasons. Generally, there are no known safety concerns with naturally occurring antioxidants as they have a Generally Regarded As Safe (GRAS) designation from regulatory authorities. Several prior studies employing an antioxidant formulation similar to the current study demonstrated antioxidant protective effects. In one study, antioxidant-pretreated mice exposed to IR, although to a high dose (2 Gy), had decreased DNA damage responses (nuclear and mitochondrial) and inflammatory responses in a variety of anatomic sites [49]. Another study involving a similar antioxidant formulation administered to patients undergoing ^99m^Tc MDP bone scans for cancer staging exhibited significantly less DSB breaks in lymphocytes [47]. Mitochondrial targeting antioxidant formulation has also been shown to result in lower oxidative stress and improved mitochondrial function in patients with mitochondrial disease [14].

The formulation of the study antioxidant consisted of multiple naturally occurring antioxidant agents as they have different actions in biological systems to reduce DNA damage from IR. Their actions include terminating free-radical chain reactions by donating electrons or hydrogen, acting as oxygen scavengers reacting and reducing oxygen, or acting as scavengers for free ROS [50]. Multiple antioxidant formulations are also more effective because of the additive effects between those with similar actions and synergistic effects of those with different modes of action [51]. Multiple antioxidants have also been shown in clinical studies to be more effective than single antioxidants in protecting against radiation-induced oxidative stress and DNA damage [42, 52]. Addition of mitochondrial-targeted antioxidants in our formulation was particularly important as mitochondria are the major source of damaging cellular ROS [53]. Effects of antioxidants on mitochondrial DNA damage repair response to IR are also important as it is at additional risk compared to nuclear DNA because it is known to be more sensitive to IR-induced oxidative stress damage, less able to repair oxidative injury, and persists longer than nuclear DNA damage [54].

### Limitations

There are several limitations with our study. Only acute short-term protective effects of antioxidants to low-dose IR were evaluated in the study. Participants had not been randomized in the study potentially creating selection bias. However, participants were also well balanced with respect to demographic factors, location, and practice setting. In addition, the baseline DNA repair biomarkers were similar between the study groups. Although participants were not blind to their intervention, the primary outcomes of the study involved quantitative measures of biomarkers unlikely affected by participants’ knowledge of the intervention. Laboratory staff were also unaware of study assignments and independently processed and assessed study biospecimens. Although the procedural fluoroscopic exposures were similar between study groups, individual dosimetry was unavailable, and scatter radiation which interventionalists are mainly exposed to may vary by various technical and behavioral factors. Despite a small sample size and heterogeneity in the DNA biomarker repair response, which has a greater risk of false negatives, statistically significant differences in repair biomarkers were found in antioxidant-treated versus untreated interventional physicians.

## Conclusions

These results show that premedication with a novel oral antioxidant formulation targeting cellular oxidative stress caused by occupationally related low-dose IR attenuated both mitochondrial and nuclear DNA damage repair response biomarkers, in peripheral lymphocytes of interventional physicians during angiography. The increase in the mRNA levels of several transcripts in the untreated control group reflected a cellular compensatory response to the deleterious effects of IR during their typical day compared to those pretreated with antioxidant supplements who had essentially no response. These findings suggest a decreased acute IR-induced oxidative stress-mediated DNA damage response.

Our study results are promising but should be considered a pilot study. Subsequent clinical studies should be conducted with larger sample sizes, across multiple institutions, and with other IR-exposed occupational groups to evaluate the generalizability of these findings. Future work should also involve long-term follow-up in order to evaluate the persistence of these effects and the oncologic protective benefit of antioxidants, which has to date been only implied.

Concern for exposure to radiation in medicine will likely continue to be of substantial clinical importance given the increasing duration and complexity of angiography interventional practices. Given the cumulative levels of radiation exposure and increased cancer risks facing healthcare professionals in this field, incorporating oral antioxidant premedication as a simple and effective strategy into a combined preventative armamentarium could offer additional protection from deleterious consequences of occupationally related IR.

## Supplementary Information

Supplementary Material 1.

Supplementary Material 2.

## Data Availability

All data generated and/or analyzed during the current study are included in the published article and its supplementary file.

## Abbreviations

*Cox4i1*: Cytochrome c oxidase subunit 4i1
IR: Ionizing radiation
mtDNA: Mitochondrial DNA
mRNA: Messenger RNA
*NF-κB*: Nuclear factor kappa-light-chain-enhancer of activated B cells
*P53*: Transformation-related protein 53
RT-PCR: Reverse transcription-polymerase chain reaction
*STAT3*: Signal transducer and activator of transcription 3
