# Supplementary material for: Antioxidant premedication reduces mitochondrial and nuclear DNA damage repair in interventional physicians performing X-ray-guided angiography procedures: a pilot clinical intervention study
Source: CVIR Endovasc. 2026 Sep 22;9:123. doi: 10.1186/s42155-026-00777-6 (PMC13597979; doi:10.1186/s42155-026-00777-6)
Supplement: Supplementary file 1 [file 42155_2026_777_MOESM1_ESM.docx]

Supplementary Material: Appendix 1: Study protocols for RNA isolation, complementary DNA (cDNA) synthesis, and reverse transcription-polymerase chain reaction analysis

**RNA isolation:** Blood samples were retrieved from -80 °C, thawed, and treated with 3-times the volume of RBC lysis buffer (ThermoFisher, J62990-AK). All samples were vortexed for 15 seconds and incubated at room temperature for 15 minutes prior to centrifugation at 2000 × g for 10 minutes at 4°C. Supernatants were discarded and pellets washed with phosphate buffered saline (Thermo Fisher Scientific, 10010023).

Samples were spun down to pellet cells and RNA was isolated using the TRIzol reagent. Briefly, upon resuspension with TRIzol, all samples were incubated at room temperature for 5 minutes. Chloroform was added to each sample, mixed vigorously for 30 s, incubated at room temperature for 3 minutes, and spun at 12,000× g for 15 min at 4 °C. Following centrifugation, the aqueous phase containing the total RNA was precipitated using isopropanol, incubated for 10 minutes at 4 °C, and centrifuged again at 12,000× g for 10 minutes at 4 °C. The resulting RNA pellet was washed with ice cold 75% ethanol and centrifuged at 7500× g for 5 minutes at 4 °C. Prior to solubilization, the pellet was air dried for 10 minutes, then re-suspended in 30 µL of RNase-free water. RNA concentration (ng/mL) and purity (260/280) were determined with the Nano-Drop 1000 Spectrophotometer (Thermo Fisher Scientific).

**Complementary DNA (cDNA) synthesis:** Reverse transcription-polymerase chain reaction (RT-PCR) analysis was a technique used to detect both mitochondrial and nuclear DNA damage and repair mechanisms from IR. Reverse transcription was performed on 1 μg cell of RNA using a SuperScript™ IV VILO™ Master Mix (Thermo Fisher Scientific, 11756500). Reaction mixtures were incubated in a T100 Thermal Cycler (Bio-Rad Laboratories) with thermocycling conditions of 25 °C for 10 minutes (annealing), 50 °C for 10 minutes (elongation), and 85 °C for 5 minutes (enzyme inactivation). Synthesized cDNA from all participants was then diluted with nuclease-free water to obtain a fixed concentration of 5.5 ng/µL prior to storage at −80 °C and subsequent RT-PCR.

**Reverse transcription-polymerase chain reaction analysis (RT-PCR):** Gene expression was determined by running samples on a CFX384 Real-Time System (Bio-Rad Laboratories). The TaqMan Fast Advanced Master Mix (Thermo Fisher Scientific, 4444557) was employed using the following TaqMan cycling conditions: 20 seconds at 95 °C for initial denaturation, 40 cycles with 3 seconds of denaturation at 95 °C, and 30 seconds of annealing and elongation at 60 °C. The fluorescence threshold was automatically set above the background level. The absence of genomic contamination was ensured using a no-template control for each probe. All RT-qPCR reactions were run in triplicates.
