# Supplementary material for: Antioxidant premedication reduces mitochondrial and nuclear DNA damage repair in interventional physicians performing X-ray-guided angiography procedures: a pilot clinical intervention study
Source: CVIR Endovasc. 2026 Sep 22;9:123. doi: 10.1186/s42155-026-00777-6 (PMC13597979; doi:10.1186/s42155-026-00777-6)
Supplement: Supplementary file 2 [file 42155_2026_777_MOESM2_ESM.docx]

Supplementary Material 2: Appendix 2. Profile of mRNA biomarkers of DNA damage repair functions

| Biomarker abbreviation | Biomarker name | DNA Damage Repair Function | mRNA marker and TaqMan probe |
| --- | --- | --- | --- |
| *P53* | Transformation-related protein 53 | Activates signaling pathways in the critical DNA damage detection and repair response pathways | Indicating nDNA damage, Hs00923894_m1 |
| *STAT3* | Signal transducer and activator of transcription | Involved in the efficient repair of DNA by modulating signaling pathways activated by P53 and involved in controlling the inflammatory process | Indicating mtDNA damage, Hs00374280_m1 |
| *NF-_K_B* | Nuclear factor kappa-light-chain-enhancer of activated B cells | A key marker of the inflammatory process | Indicating inflammation, Hs00765730_m1 |
| *COX4i1* | Cytochrome c oxidase subunit 4i1 | This nuclear encoded sub-unit of the mitochondrial respiratory chain is a major component of oxidative phosphorylation and is coordinately upregulated with mitochondrial DNA (mtDNA) replication to allow for mitochondrial biogenesis for cells to respond to DNA damage and maintain genetic stability | Indicating mitochondria**l** biogenesis, Hs00971639_m1 |
